# Accumulation of Salicylic Acid and Related Metabolites in *Selaginella moellendorffii*

**DOI:** 10.3390/plants11030461

**Published:** 2022-02-08

**Authors:** Anna Berim, David R. Gang

**Affiliations:** Institute of Biological Chemistry, Washington State University, Pullman, WA 99164, USA; gangd@wsu.edu

**Keywords:** salicylic acid, dihydroxybenzoic acid, phenylpropanoic acids, metabolism, *Selaginella moellendorffii*

## Abstract

Salicylic acid (SA) is a phytohormone that plays manifold roles in plant growth, defense, and other aspects of plant physiology. The concentration of free SA in plants is fine-tuned by a variety of structural modifications. SA is produced by all land plants, yet it is not known whether its metabolism is conserved in all lineages. *Selaginella moellendorffii* is a lycophyte and thus a representative of an ancient clade of vascular plants. Here, we evaluated the accumulation of SA and related metabolites in aerial parts of *S. moellendorffii*. We found that SA is primarily stored as the 2-*O*-*β*-glucoside. Hydroxylated derivatives of SA are also produced by *S. moellendorffii* and stored as *β*-glycosides. A candidate signal for SA aspartate was also detected. Phenylpropanoic acids also occur in *S. moellendorffii* tissue. Only *o*-coumaric acid is stored as the *β*-glycoside, while caffeic, *p*-coumaric, and ferulic acids accumulate as alkali-labile conjugates. An in silico search for enzymes involved in conjugation and catabolism of SA in the *S. moellendorffii* genome indicated that experimental characterization is necessary to clarify the physiological functions of the putative orthologs. This study sheds light on SA metabolism in an ancestral plant species and suggests directions towards elucidating the underlying mechanisms.

## 1. Introduction

The genus *Selaginella* belongs to the class Lycopodiopsida, a clade of non-seed vascular plants that diverged from euphyllophytes about 400 million years ago [1,2]. Within the genus that counts over 700 species [3], the genome of *S. moellendorffii* was sequenced several years ago [4], positioning it as an emerging model species for investigations into plant evolution [5], including the evolution of plant metabolism [6]. More recently, genomes of the desiccation-tolerant *S. tamariscina* [7] and *S. lepidophylla* [8] have been sequenced as well.

Even prior to genome sequencing, *S. moellendorffii* was subject to comparative studies of its lignin biosynthesis, which revealed some examples of lineage-specific processes [9,10]. Furthermore, phytochemical analyses of various *Selaginella* species revealed the accumulation of both common specialized metabolites and complex compounds unique to this genus, such as selaginellins (for review, see ref. [6]).

Salicylic acid (SA) is a multi-faceted phytohormone with a prominent role in plant defense [11,12]. Numerous studies indicate it is critical for establishing systemic acquired resistance [13,14]. The physiological functions and effects of SA range from response to biotrophic pathogens to involvement in flowering, thermogenesis, and senescence [11,15]. All land plant lineages appear to be able to synthesize SA and possess SA signaling elements [16,17], suggesting its function as a defense hormone may be conserved. SA was also detected in the charophyte *Klebsormidium flaccidum* [18], yet the analysis of several available charophyte genomes suggested SA signaling is not functional in this clade [16,17]. Free SA content is fine-tuned both at the level of biosynthesis and catabolism. In many plants, basal or pathogen-induced SA is stored as the inactive 2-*O*-glucoside (SAG) [11,12]. An additional, less abundant glucose conjugate is the SA glucose ester (SGE) [15]. Methylation of the carboxylic acid results in methyl salicylate (MeSA) [12]. MeSA is discussed as a mobile defense signal [19,20], and its basal abundance is low [21]. Furthermore, SA can be conjugated with aspartic acid to form *N*-saliciloyl aspartate [11,12]. A sulfotransferase capable of sulfonating SA was identified in Arabidopsis, yet the occurrence of sulfonated SA conjugates was not detected [22]. Two of the described SA catabolic pathways involve hydroxylations at position 3 or 5 of the benzenoid ring, resulting in 2,3- and 2,5-dihydroxybenzoic acids (DHBAs) [23,24]. Arabidopsis lines with altered levels of these DHBAs exhibit pronounced phenotypes such as changed susceptibility to downy mildew and early senescence. These phenotypes are primarily attributed to the changed SA levels in those lines [23]. 2,3- and 2,5-DHBA are subsequently glycosylated [23,25,26]. There is evidence that glycosylated DHBAs modulate the immune responses in Arabidopsis [25,27], tomato, and cucumber [26].

The purpose of this exploratory study was to assess SA metabolism in a representative of Lycopodiopsida. Currently, there is no information regarding the accumulation of SA and its metabolites in the genus *Selaginella*. To close this gap in knowledge, we evaluated the occurrence of SA and its derivatives, selected related benzenoids, and phenylpropanoic acids in aqueous-methanolic extracts from the aerial tissue of *S. moellendorffii*. Metabolite abundance in these untreated extracts was compared to levels in extracts subjected to hydrolysis and subsequent extraction with ethyl acetate. Three types of hydrolysis were conducted: enzymatic *β*-glycoside cleavage with almond glucosidase, alkaline hydrolysis that is expected to selectively cleave esters, and full chemical hydrolysis comprising treatment with both alkali and acid that would cleave esters, glycosides, and potentially other conjugates of SA and other analyzed acids. To make predictions regarding the mechanisms of SA modifications, the *S. moellendorffii* genome was searched for candidate orthologs of proteins catalyzing SA hydroxylation, glycosylation, and amino acid conjugation in flowering plants. An analysis of SA and its metabolites in *S. moellendorffii* provides insight into the evolution of these branches of plant defense in this ancient lineage.

## 2. Results and Discussion

### 2.1. Occurrence of Salicylic Acid and Its Storage Forms

**Figure 1:**
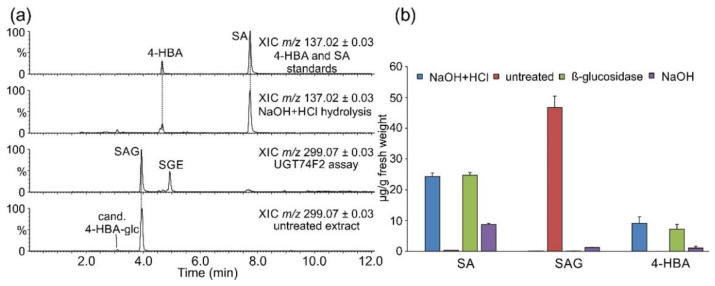
Accumulation of salicylic acid (SA), salicylic acid glucoside (SAG), and 4-hydroxybenzoic acid (4-HBA). Metabolites were separated and analyzed by LC-UV-MS, as described in Section 3. (**a**) Selected ion chromatograms. Displayed *m*/*z* and mass window, and the identity of the sample is indicated for individual traces. (**b**) Concentrations in differently treated extracts. Data are presented as the average of technical duplicates of three biological replicates, and error bars show standard deviation.

SAG was readily detectable in untreated samples, while the levels of free SA in untreated samples were very low. SA was present at very similar levels in *β*-glucosidase-treated and chemically fully hydrolyzed samples (Figure 1). Storage of SA as SAG in *S. moellendorffii* is similar to observations in numerous other species [11]. The observed total SA levels are similar to those found in Arabidopsis [23]. Several species such as rice [28,29], potato [30], and soybean [23] were reported to accumulate much higher amounts of total and free SA, raising questions regarding the perception and role of SA in these species. It should also be mentioned that there is a considerable degree of variation regarding SA concentration in the same species as reported in different studies, e.g., Arabidopsis [23,24,25,31]. Therefore, experiments involving direct comparison across species may present a more reliable source of such information [23].

**Figure 2:**
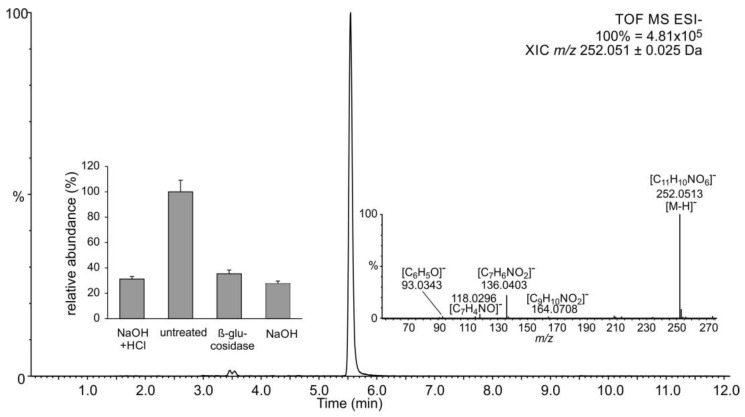
Detection of candidate SA-Asp. Metabolites were separated and analyzed by LC-UV-MS, as described in Section 3. Selected ion chromatogram at the expected mass-to-charge ratio in negative ion mode is shown. Insets show the MS*^E^* spectrum annotated with predicted elemental compositions of relevant fragments and relative abundance of the signal in differently treated extracts. Additionally, 100% corresponds to 2.12 × 10^6^ arbitrary units (peak area under the curve). Data are presented as the average of technical duplicates of three biological replicates, and error bars show standard deviation.

Alkaline-only hydrolysis at room temperature does not affect glycosides but efficiently cleaves glucose esters of phenylpropanoic and benzoic acids. Even though no SGE was detected in untreated extracts (Figure 1a), SA was present in alkaline-hydrolyzed samples at ca. 30% of levels observed in samples treated with both acid and base (Figure 1b). This suggests that some SA accumulates as a different, not previously reported alkali-labile conjugate. It is also possible, although unreported, that SAG partially hydrolyzes under treatment with sodium hydroxide applied in this study. Data were also surveyed for the presence of *N*-saliciloyl aspartate (SA-Asp) that was previously found in several species [31,32,33,34]. A candidate signal, supported both by accurate precursor mass and fragmentation, was present in untreated samples as well as in those from all treatments at a lower abundance (Figure 2).

Amides of amino acids are fairly resistant to chemical hydrolysis, so it is conceivable that at least some SA-Asp would remain intact and get extracted into the ethyl acetate layer under applied treatments. Importantly, an aspartate conjugate with 4-hydroxybenzoic acid (4-HBA) as the acyl residue would produce the same mass spectrum. A comparison with an authentic standard(s) is necessary for unambiguous clarification of the signal’s identity. SA-Asp is thought to be an inactive storage form of SA in Arabidopsis [34], while an aspartate amide of 4-HBA has not been reported to date.

### 2.2. Occurrence of Related Benzoic Acids

**Figure 3:**
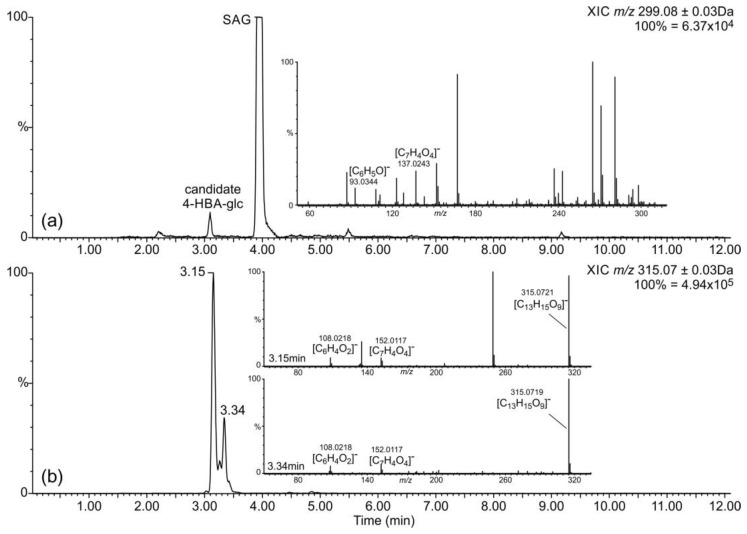
Detection of candidate 4-HBA hexoside and DHBA hexosides. Metabolites were separated and analyzed by LC-UV-MS, as described in Section 3. Selected ion chromatograms of mass-to-charge ratios matching those expected for (**a**) 4-HBA hexoside (same ion as SAG, same trace as in Figure 1, zoomed in for better visibility) and (**b**) DHBA hexoside, as observed in untreated extracts. Insets show the MS*^E^* spectra annotated with predicted elemental compositions of relevant fragments.

SA homeostasis is in part maintained by its conversion into dihydroxylated derivatives 2,3- and 2,5-DHBA [11]. Additionally, we wanted to evaluate the occurrence of other simple benzenoids such as 4-HBA in *S. moellendorffii*. Similar levels of 4-HBA were detected in *β*-glucosidase-treated and chemically hydrolyzed samples (Figure 1b). A candidate signal for 4-HBA hexoside was present in the untreated samples (Figure 1 and Figure 3a). 4-HBA occurs in small amounts in all plants as an intermediate of ubiquinone biosynthesis [35] and has been detected in *Selaginella* species [6]. It also accumulates at higher levels in plants where it serves as an intermediate in other pathways, e.g., in shikonin biosynthesis [36]. An increase in 4-HBA and SA levels occurred in cucumber phloem upon *Pseudomonas* infection [37], yet a concrete role for 4-HBA in defense has not been well defined.

**Figure 4:**
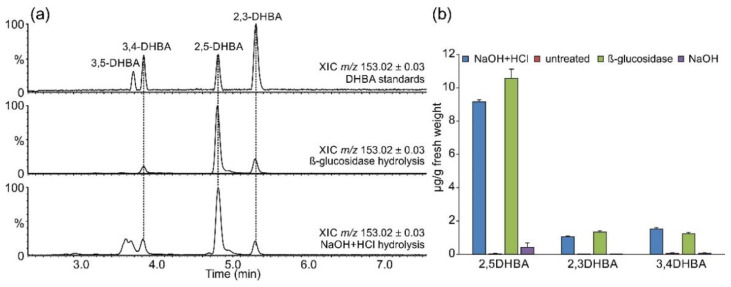
Accumulation of DHBAs. Metabolites were separated and analyzed by LC-UV-MS, as described in Section 3. (**a**) Selected ion chromatograms. Displayed *m*/*z* and mass window, and the identity of the sample is indicated for individual traces. (**b**) Concentrations in differently treated extracts. Data are presented as the average of technical duplicates of three biological replicates, and error bars show standard deviation.

Several DHBAs were readily detectable in glucosidase-treated and chemically hydrolyzed samples (Figure 4). As observed for SA and 4-HBA, the aglycon levels were comparable in these two preparations, suggesting that *β*-glycosides represent the major accumulated form. Comparison with authentic standards revealed the identities of the two less abundant signals as 3,4- and 2,3-DHBAs. It is worth mentioning that under the applied chromatographic conditions, 2,6-DHBA and especially 2,4-DHBA eluted at retention times very similar to those of 2,3-DHBA. The identity of the corresponding peak in *S. moellendorffii* extracts as 2,3-DHBA was ascertained by co-chromatography. The strongest DHBA signal corresponded to gentisic acid (2,5-DHBA). A fourth signal with the retention time close to that of 3,5-DHBA and the accurate mass of a DHBA was observed in chemically hydrolyzed samples but not in other samples. However, it lacked the characteristic *m*/*z* 109.0290 fragment corresponding to decarboxylated DHBA [C_6_H_6_O_2_-H]^-^ ion and must originate from another compound.

Two signals with accurate masses matching DHBA hexosides were present in untreated extracts (Figure 3b), but no candidate DHBA xylosides were found. DHBA xylosides were previously reported from Arabidopsis [25] as well as tomato and cucumber [26]. Notably, a survey of a number of flowering plants suggested that gentisic acid is more widely spread in the plant kingdom than 2,3-DHBA, at least in the ten studied dicot plants that were analyzed at two developmental stages: young and senescent [23]. The levels of gentisic acid found in *S. moellendorffii* in this study were within the range observed in those dicot species, while the levels of 2,3-DHBA were considerably lower than those found in the two accumulating species, Arabidopsis and *Vinca major* [23].

**Figure 5:**
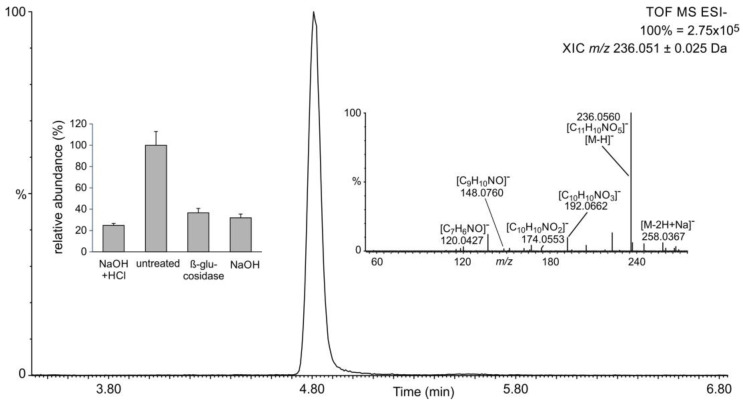
Detection of candidate BA-Asp. Metabolites were separated and analyzed by LC-UV-MS, as described in Materials and Methods. Selected ion chromatogram at the expected mass-to-charge ratio in negative ion mode is shown. Insets show the MS*^E^* spectrum, annotated with predicted elemental compositions of relevant fragments, and relative abundance of the signal in differently treated extracts. Additionally, 100% corresponds to 1.04 × 10^6^ arbitrary units (peak area under the curve). Data are presented as average of technical duplicates of three biological replicates, and error bars show standard deviation.

Upon detecting a strong candidate signal for SA-Asp, the data were also searched for benzoyl *N*-aspartate (BA-Asp). A plausible candidate signal was strongest in the untreated samples but also present in all extracts that underwent hydrolysis, albeit at lower levels (Figure 5). Its retention time is longer than that of candidate SA-Asp (Figure 2 and Figure 5); however, under the chromatographic conditions used, the retention time of SA was also longer than that of BA, potentially due to an intramolecular hydrogen bond between the 2-OH and the carboxylic moiety. BA-Asp was previously detected in pea seeds [38] and formed after feeding benzoic acid to *Lemna paucicostata* [39]. Furthermore, Westfall et al. [31] found that BA-Asp accumulated in Arabidopsis leaves alongside SA-Asp. The enzyme catalyzing the formation of SA-Asp is also capable of producing BA-Asp [31].

### 2.3. Occurrence of Phenylpropanoic Acids

**Figure 6:**
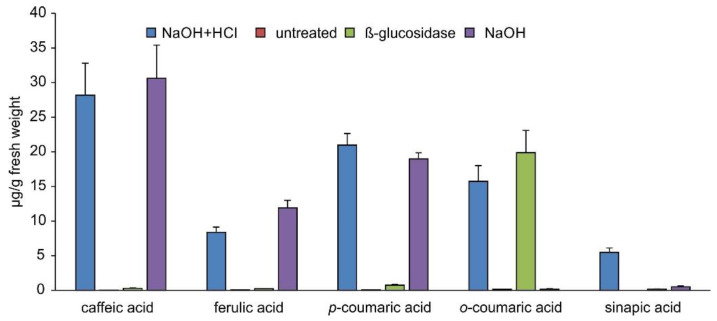
Abundance of phenylpropanoic acids in different extracts. Data are presented as average of technical duplicates of three biological replicates, and error bars show standard deviation.

The metabolism of phenylpropanoic acids is connected with benzenoid metabolism [40]. We, therefore, evaluated the abundances of the main phenylpropanoic acids in extracts from *S. moellendorffii*. Chemically fully hydrolyzed samples contained significant amounts of caffeic, *p*-coumaric, *o*-coumaric, ferulic, and sinapic acids (Figure 6). Caffeic, *p*-coumaric, and ferulic acids were also released by alkaline hydrolysis. In contrast, extracts that have been subjected to glucosidase treatment only contained *o*-coumaric acid at levels similar to those observed after chemical hydrolysis. Sinapic acid was not very abundant in all other samples.

**Figure 7:**
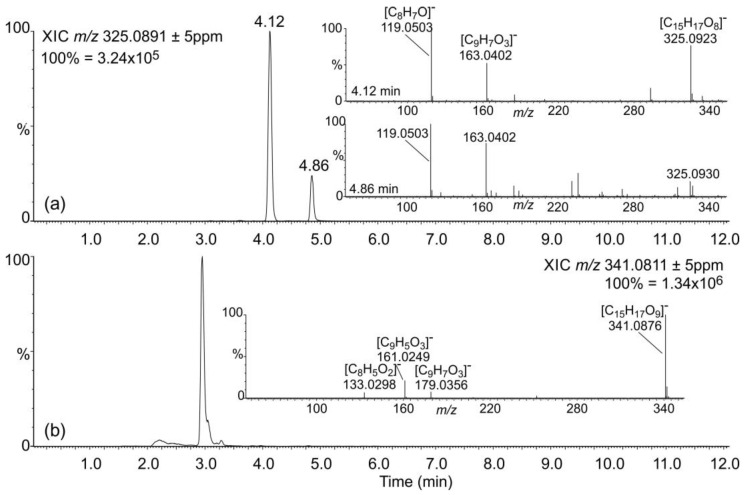
Detection of candidate phenylpropanoic acid conjugates. Metabolites were separated and analyzed by LC-UV-MS, as described in Section 3. Selected ion chromatograms for candidate C_6_H_12_O_6_ conjugates with coumaric (**a**) and caffeic (**b**) acids in untreated extracts are presented. Insets show MS*^E^* spectra annotated with predicted elemental compositions of relevant fragments.

**Figure 8:**
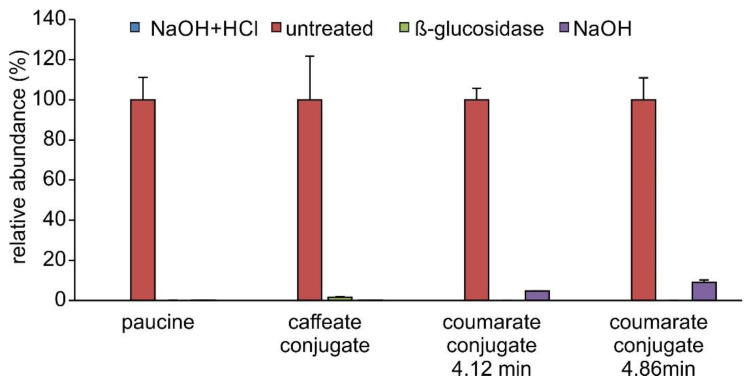
Relative abundances of paucine and C_6_H_12_O_6_ conjugates of caffeic and coumaric acids. Abundances observed in different extracts are shown as % of signal in the extract with the highest abundance, the average of which is set to 100%, and equals 6.56 × 10^6^ (paucine), 7.65 × 10^6^ (caffeate conjugate), 1.82 × 10^6^ (coumarate conjugate at 4.12 min), and 4.84 × 10^5^ (coumarate conjugate at 4.86 min) arbitrary units (peak area under the curve). Data are presented as the average of technical duplicates of three biological replicates, and error bars show standard deviation.

These findings suggested that caffeic, *p*-coumaric, and ferulic acids do not accumulate as *β*-glycosides but rather as esters or some other alkali-labile conjugates in *S. moellendorffii*. Untreated extracts were searched for candidate signals for these compounds. *S. moellendorffii* has been reported to contain several *myo*-inositol caffeates [41]. Phenylpropanoic acids are also known to accumulate as glucose esters in plants [42]. However, the latter can reportedly be cleaved by almond *β*-glucosidase [15,43], which would not fit the abundance profile observed in this study (Figure 6). The elemental composition of *myo*-inositol is the same as that of glucose; hence, such conjugates would not be readily distinguishable by mass spectrometry. One signal corresponding to candidate *myo*-inositol caffeate and two signals corresponding to potential C_6_H_12_O_6_ conjugates with coumaric acid were readily detectable (Figure 7) and most abundant in untreated samples (Figure 8). One of the latter two signals may represent the *β*-glycoside of *o*-coumaric acid. However, no promising candidate signal for the feruloyl ester with a C_6_H_12_O_6_ moiety was observed. The storage of *o*-coumaric acid as 2-*O*-glucoside is well known from studies into coumarine biosynthesis [44,45]. Early studies discussed *o*-coumaric acid as a potential intermediate of SA biosynthesis [46], yet more recent investigations pointed to other routes [15]. It is also possible that these pathways are redundant or vary between species.

An analysis of substrate preferences of CYP98A34, the *S. moellendorffii* homolog of *p*-coumarate 3′-hydroxylases, revealed that it is most active with *p*-coumaroyl anthranilate [47]. Data were, therefore, searched for anthranilate conjugates of coumaric and caffeic acid, but no pronounced candidate signals were observed. In line with substrate preferences of CYP98A34 [47], no promising candidate signal was found for caffeoyl quinic, i.e., chlorogenic acid.

A very strong signal corresponding to paucine, the putrescine conjugate of caffeic acid, was present in untreated samples, in agreement with previous reports of its occurrence in *S. moellendorffii* [41]. It was absent from all of the treated samples (Figure 8). This behavior may be explained by the protonation of the primary amino residue of putrescine, which would prevent the charged product from being extracted into ethyl acetate. A search for analogous conjugates of ferulic and coumaric acids did not return potential hits. Untreated samples also contained a candidate signal for coumaroyl agmatine, another polyamine conjugate reported from *S. moellendorffii* [41]. The signal was ca. 200-fold weaker than that for paucine. As observed for paucine and possibly for the same reasons, it was absent in all treated samples.

### 2.4. Candidate SA-Modifying Proteins in S. moellendorffii Genome

In Arabidopsis, the biosynthesis of 2,5-DHBA and 2,3-DHBA is catalyzed by oxoglutarate-dependent dioxygenases (ODDs) Downy Mildew Resistant 6 (DMR6) and DMR6-like 1 (DLO1), respectively [23,24]. Notably, DLO1 catalyzes both 3- and 5-hydroxylation of SA in vitro [24]. DMR6 and DLO1 proteins share 50% identity. A second DMR6-like protein, DLO2, sharing 75% identity with DLO1, is present in the Arabidopsis genome but exhibits a different expression pattern than DLO1 and has not been biochemically characterized [48]. The *S. moellendorffii* RefSeq genome comprises >140 ODD-like sequences that are found by BLAST search on the NCBI server. Twenty of those are annotated as “DMR6-like” or “DMR6” proteins. The alignment of those proteins revealed that several of them are redundant or very similar to each other. This is an expected observation as the sequenced genome covers two haplotypes with an overall identity of 98.5% at the nucleotide level [4]. BLAST back searches against the NCBI database showed that DMR6, DLO1, and DLO2 are indeed the top experimentally characterized matches for those *S. moellendorffii* proteins (Table 1). The identities with Arabidopsis ODDs were below 45% in all cases, which is not unexpected given the evolutionary distance. However, in most cases, the difference between the identity levels and alignment scores with DMR6 and DLO1 was very small. Experimental functional characterization is therefore necessary to identify the true SA 3- and 5-hydroxylases in *S. moellendorffii*. An earlier analysis of the ODD protein family in *S. moellendorffii* showed that it contained 74 members [49]. Eight belonged to the ancient DOXA class that is involved in DNA repair and seven to the DOXB clade that comprises proline-4-hydroxylases. Only nineteen ODDs of the DOXC class that is involved in specialized metabolism clustered together with proteins from other co-analyzed plants, but none was assigned to cluster DOXC38, which harbors Arabidopsis SA hydroxylases. Twenty-six *S. moellendorffii* ODDs clustered with each other, forming eight species-specific groups [49].

The above results prompted us to evaluate the conservation of known SA hydroxylases in well-studied plant species. A search for Arabidopsis DMR6 homologs in the NCBI RefSeq database under exclusion of models (XP) resulted in sequences with >65% identity from other dicotyledonous species such as *Vitis vinifera*, *Nicotiana tabacum*, and *Solanum lycopersicum*. Knockout mutants of DMR6 ortholog in *S. lycopersicum* display broad pathogen resistance [50]. Similarly, near-complete knockout mutants of DMR6 in *Ocimum basilicum* displayed enhanced resistance to downy mildew [51]. A 60% identical homolog is present in the monocot *Zea mays*. That protein possesses flavone synthase activity [52] and is a mildew susceptibility factor [52,53]. Its SA 5-hydroxylase activity has not been assessed. Cumulatively, this evidence supports a conserved role for DMR6 in pathogen resistance in angiosperms. An analogous search for homologs of SA 3-hydroxylase indicated it is less conserved, with the highest identities found in the NCBI RefSeq database (excluding models) being below 61%. Notably, the top match was a protein from maize annotated as DLO1. To the best of our knowledge, its function has not been experimentally determined.

The biosynthesis of SA-Asp is catalyzed by GH3.5, an amidotransferase of the GH (Gretchen Hagen) 3 family [31,54,55,56], which is also capable of producing BA-Asp and contributes to its accumulation in Arabidopsis [31]. GH3.5 is involved in several regulatory processes with a primary role of conjugating the auxin indole acetic acid (IAA) to aspartate [55,56,57]. IAA-Asp is subsequently catabolized. Recently, another enzyme of this family, GH3.12, was shown to catalyze the penultimate step in SA biosynthesis via the isochorismic acid pathway, namely the conjugation of isochorismic acid to glutamate [58,59]. GH3.12 has initially been characterized as an enzyme conjugating glutamate with 4-HBA and 4-aminobenzoic acid [60]. A BLAST search of the *S. moellendorffii* RefSeq genome using GH3.5 from Arabidopsis as query resulted in 36 hits overall. The top match exhibited 66.4% identity with the query (Table 1). A redundant second match was 99.5% identical with the first. The same two sequences were the top matches for a BLAST search using GH3.12 from Arabidopsis as a query, with 46.9% identity. The next best matches had somewhat lower identity levels (≤43%) and alignment scores but matched some other GH3 proteins from Arabidopsis better than GH3.12. Two sequences differing by a 17-amino acid stretch that is missing from one of the sequences were automatically annotated as GH3.12 isoform X1 (XP_024543479.1) and X2 (XP_002961596.1) but were shorter than 450 amino acids and appeared to be incomplete. Notably, searches for GH3.12 homologs within the angiosperms indicated that this amidosynthetase is less conserved than GH3.5. For example, the best match for Arabidopsis GH3.12 from *Solanum* was 50% identical with the query. By contrast, a BLAST search for *Solanum* homologs of Arabidopsis GH3.5 returned hits with 80% identity. Auxin biosynthesis and signaling evolved in charophytes [16] and are indispensable for plant growth and development [61]. The critical role of auxins may be one of the factors explaining the stronger conservation of auxin-modifying proteins throughout the evolution of plants.

Several UDP-glucose-dependent glycosyltransferases (UGTs) from Arabidopsis catalyze the formation of SGE and/or SAG. UGT74F1 forms only SAG, while UGT74F2, which is 76.84% identical to UGT74F1, produces both SGE and SAG [62] in vitro. In addition, UGT76B1 is capable of producing SAG, while its primary role is to convert another plant signal molecule, *N*-hydroxypipecolic acid, into the corresponding glucoside [63,64,65,66]. UGT76B1 shares less than 30% identity with UGT74F proteins. A recent phylogenomic analysis of UGTs in sequenced plant genomes identified 137 putative UGTs in *S. moellendorffii*, all of which clustered separately from the UGTs of seed plants [67]. Experimental functional information regarding Selaginella UGTs is currently scarce aside from a study that evaluated the glycosyltransferases of *S. moellendorffii* involved in cell wall biosynthesis [68]. A targeted BLAST search of the *S. moellendorffii* RefSeq genome using any of the three SA-modifying Arabidopsis UGTs as query returned 211-212 hits with identities not exceeding 35% and alignment scores below 220. Back searches of the top matches did not return SA-modifying UGTs as best hits (Table 1). Functional analysis of the best candidates is necessary to identify the enzyme(s) responsible for the accumulation of SAG. A review of available data supported the notion that SA glycosyltransferases are not well conserved across the plant kingdom or even within the angiosperms. In well-characterized genomes, the closest homologs of Arabidopsis UGT74F1 and UGT76B1 are less than 50% identical (e.g., in tobacco, tomato, and potato). A characterized rice OsSGT1 is 46% and 44% identical to UGT74F1 and UGT74F2, respectively, and catalyzes only the formation of SAG [69]. Very recently, a novel protein termed UGT87E7 that selectively forms SGE was identified in *Camelia sinensis* [70]. Remarkably, downregulation of the underlying gene’s expression results in decreased accumulation of both SGE and SA and increased susceptibility to pathogens, suggesting that its products play a positive role in the disease resistance of this species. Overall, the divergence of SA-modifying UGTs underscores their role as regulators and modulators of SA’s biological activity.

## 3. Materials and Methods

### 3.1. Plant Material

Three plants of *S. moellendorffii* were obtained from Plant Delights Nursery, Raleigh, NC, USA. They were potted in Sungro #1 potting soil mix and maintained in the greenhouse under a humidity dome with ca. 80% relative humidity and a 16h day with supplemental lighting of ca. 100 μmol·m^−2^·s^−1^ and 18 °C (night)/24 °C (day) regimen. Several shoots were collected from each plant and extracted for analysis.

### 3.2. General Chemicals

All solvents used for extraction were of HPLC grade or better. LC-MS grade solvents were from Millipore Omnisolv (MilliporeSigma, Burlington, MA, USA). Authentic standards of all benzoic (SA, 4-HBA, 2,3-, 2,4-, 3,5-, 3,4-, 2,6-DHBA) and phenylpropanoic (caffeic, *o*-, *m*-, *p*-coumaric, ferulic, sinapic) acids were from Sigma. Authentic SAG was purchased from Santa Cruz Biotechnology, Inc. (Dallas, TX, USA).

### 3.3. Extract Preparation

Fresh aerial tissue (ca. 5–7 cm long tips of shoots comprising stems and microphylls) was flash-frozen in liquid nitrogen and ground in a ball mill (Tissuelyser II, Qiagen, Hilden, Germany) for 1 min at 30 s^−1^. The extraction method was modified after ref. [23]. Frozen ground tissue was extracted with 90% aq. methanol at a ratio of 1 mL solvent per ca. 100 mg tissue by sonicating it for 5 min (40 Hz, 22 °C, Branson 5510 sonication bath, Branson Ultrasonics Corp., Brookfield, CT, USA) and subsequent incubation at 4 °C for 1 h under slow agitation. Extracts were clarified by centrifugation, and supernatants were transferred to a new tube. The resulting pellets were re-extracted using the same procedures. Combined extracts were supplemented with 1 μM internal standard 4-bromo-3,5-dihydroxybenzoic acid (Sigma-Aldrich, St. Louis, MO, USA), divided into aliquots of 1 mL, and dried under vacuum. Eight aliquots were prepared for each biological replicate. They were used to prepare duplicates of four types of samples: untreated (unhydrolyzed) extracts, enzymatically hydrolyzed extracts, extracts subjected to alkaline (NaOH) hydrolysis only, and extracts subjected to both alkaline and acid (NaOH + HCl) hydrolysis. Two aliquots were kept frozen for later analysis as untreated extracts. Two aliquots for enzymatic hydrolysis were suspended in 290 μL 100 mM sodium acetate, pH 5.0, containing 1 mg·mL^−1^ *β*-glucosidase from sweet almonds (1000 U·mg^−1^, MP Biomedicals cat.-no 100348), and incubated at 37 °C for 16 h. The remaining four aliquots for non-enzymatic hydrolysis were suspended in 100 μL 1 N sodium hydroxide and incubated at ambient temperature for 16 h. Thereafter, 200 μL 6 N HCl were added to two of the alkaline-treated aliquots, which were then incubated at 80 °C for 1 h. The other two alkaline-treated aliquots were also acidified with 200 μL 6 N HCl, then immediately extracted with 350 μL ethyl acetate. Samples undergoing acid hydrolysis were first allowed to cool, then extracted with ethyl acetate. Glucosidase-treated samples were acidified with 20 μL 6 N HCl prior to extraction with ethyl acetate. After centrifugation to remove the organic layer, all 6 hydrolyzed samples were re-extracted with the same volume of ethyl acetate. Combined first and second ethyl acetate fractions were dried in vacuum. To avoid losses of SA through sublimation, the drying process was closely monitored and terminated as soon as solvent was eliminated. For LC-MS analysis, the dry residues for untreated samples and the dry residues after hydrolysis and ethyl acetate extraction were suspended in 100 μL 50% aq. methanol containing 0.1% formic acid, dissolved by vigorous mixing and brief sonication, and clarified by centrifugation prior to transfer to LC-MS vials.

### 3.4. Analysis by UPLC-PDA-qTOF-MS

A Synapt G2-S HDMS quadrupole time-of-flight mass spectrometer system (Waters, Waters Corp., Milford, MA, USA) equipped with an Acquity UPLC system with a photodiode array detector was used for LC-MS analysis. Analytes were separated on an Acquity HSS T3 UPLC column (100 × 2.1 mm, particle size 1.8 μm) using the linear gradient described by Bartsch et al. [25]. Two microliters of sample were loaded onto the column. The column temperature was kept at 40 °C while the samples were at 8 °C. Mass spectra were collected in negative continuum electrospray ionization mode over a range of *m*/*z* 50–1200 with a scan time of 0.2 s. The capillary was at 2.5 kV, the sampling cone was at 40 V, and the source was at 100 °C, with the desolvation temperature at 250 °C; cone gas and desolvation gas flow were at 0 and 850 L·h^−1^, respectively. Fragmentation was carried out in a data-independent mode using a collision energy ramp of 15–50 eV. The mass spectrometer was calibrated using sodium formate with a 95% confidence cutoff of 1 ppm. Leucine enkephalin was infused throughout the run and used for post-acquisition mass correction. Data analysis was performed using MassLynx 4.1 and integrated modules (Waters). To facilitate more broadly targeted evaluation, data were additionally processed by Progenesis QI 2.4 (Waters). UV data were collected over a range of 210–500 nm. Data were normalized to the weight of extracted tissue and the internal standard (4-bromo-3,5-dihydroxybenzoic acid). For the absolute quantification of SA, SAG, 4-HBA, 2,3-DHBA, 2,5-DHBA, caffeic, *p*-coumaric, *o*-coumaric, and sinapic acids, external 6-point calibration curves were constructed using serial dilutions of authentic standards (1–50 μM). Where necessary, extracts were diluted and reanalyzed to fit the calibration range. Data are presented as averages of technical duplicates of 3 biological replicates; error bars show standard deviation.

### 3.5. Search for Candidate SA-Modifying Proteins

For SA hydroxylases, Arabidopsis SA3H (NP_192788.1, AT4G10500) and SA5H (NP_197841.1, AT5G24530) were used as queries to BLASTP search the NCBI Refseq database (release 208 from 13 September 2021) for *Selaginella moellendorffii*. Arabidopsis GH3.5 (NP_194456.1, AT4G27260) and GH3.12 (NP_196836.1, AT5G13320) were used as query for amidotransferases. Candidate SA glycosyltransferases were retrieved using UGT74F1 (NP_973682.1, AT2G43840) and UGT76B1 (NP_187742.1, At3G11340) as queries. Default BLAST settings were applied, and the maximum hit number was set to 250. For each protein type (ODD, GH3, or UGT), *S. moellendorffii* hits were aligned using Clustal Omega [71], and identity matrices were used to identify sequences with very high identity levels. Non-redundant hits were used as queries for back BLAST searches against the NCBI GenBank non-redundant protein database or against the Arabidopsis reference protein database.

### 3.6. Enzymatic Production of Authentic SGE

Recombinant UGT74F2 protein was produced as described previously [72] and incubated with UDP-glucose and SA to generate an authentic retention time standard for SGE. Assays were analyzed as described in Section 3.4.

## 4. Conclusions

The present analysis of aerial parts of *S. moellendorffii* revealed that this ancestral species accumulates the same metabolites of SA as angiosperms. As observed in other species, the content of free SA is low, and it is predominantly stored as its 2-*O*-*β*-glucoside SAG. A significant portion of SA is hydroxylated to yield gentisic acid, while the branch of metabolism converting it into 2,3-DHBA is somewhat less active. A candidate signal for the conjugate of SA with aspartate was also detected. In silico search for potential catalysts underlying those transformations indicated that functional analysis of candidate proteins is necessary to demonstrate their physiological functions. This future investigation will reveal whether the mechanisms of SA modifications are conserved or have evolved independently several times throughout plant evolution.

## Figures and Tables

**Table 1:** In silico search for SA-modifying proteins.

		Top BLAST NR Proteins		Top BLAST AraTh RefSeq		Top BLAST Biochemically Characterized AraTh RefSeq Protein	
Protein Type	SmRefSeq	Species and Accession Number	%ID/Score	Accession Number (Gene Name if Any)	%ID/Score	Accession Number (Gene Name if Any)	% ID/Score
ODD	XP_024534841.1	*Picea sitchensis* ABK26685.1	47.46/338	NP_192787.1 (DLO2)	43.88/295	NP_192788.1 (DLO1)	43.60/294
ODD	XP_002968368.2	*Pinus tabuliformis* GA2ox11 AHW42461.1	51.61/363	n/a ^1^		NP_192788.1 (DLO1)	47.14/285
ODD	XP_024525160.1	*Picea sitchensis* ABK22784.1	48.14/312	NP_192787.1 (DLO2)	41.71/278	NP_192788.1 (DLO1)	42.42/272
ODD	XP_002965938.2	*Pinus tabuliformis* GA2ox11 AHW42461.1	53.22/373	n/a		NP_192788.1 (DLO1)	43.54/281
ODD	XP_002968370.1	*Pinus tabuliformis* GA2ox11 AHW42461.1	51.17/359	n/a		NP_192788.1 (DLO1)	47.60/284
ODD	XP_002967783.1	*Aquilegia coerulea* PIA25996.1	32.25/204	n/a		NP_192788.1 (DLO1)	33.43/182
ODD	XP_002988299.1	*Musa acuminata* XP_009389864.1	39.45/233	n/a		NP_197841.1 (DMR6)	37.61/211
ODD	XP_002983067.1	*Marchantia polymorpha* PTQ39739.1	39.63/251	NP_192787.1 (DLO2)	38.87/210	NP_197841.1 (DMR6)	39.26/207
ODD	XP_002975060.1	*Tetracentron sinense* KAF8411241.1	41.77/263	NP_192787.1 (DLO2)	37.61/234	NP_197841.1 (DMR6)	37.73/227
GH3	XP_024536354.1	*Ricinus communis* XP_002533739.1	68.07/879	n/a		NP_194456.1 = GH3.5/WES1	66.4/862
GH3	XP_002960824.1	*Brassica carinata* KAG2310584.1	48.99/592	n/a		NP_200262.1 = GH3.6/DFL1	48.55/586
GH3	XP_024519979.1	*Daucus carota* subsp. sativus XP_017250111.1	45.02/504	n/a		NP_200262.1 = GH3.6/DFL1	43.39/480
GH3	XP_024529423.1	*Vigna angularis* XP_017432135.1	43.77/503	n/a		NP_200262.1 = GH3.6/DFL1	43.01/488
GH3	XP_024516808.1	*Punica granatum* XP_031407799.1	39.58/415	NP_001319858.1 GH3.10/DFL2	36.80/393	NP_566071.1 GH3.11/JAR1/FIN219	36.54/380
GH3	XP_002976207.1	*Ceratopteris richardii* KAH7428824.1	39.89/412	n/a		NP_566071.1 GH3.11/JAR1/FIN219	36.36/378
GH3	XP_024540069.1	*Tanacetum cinerarifolium* GEY79194.1	42.33/501	NP_001319858.1 GH3.10/DFL2	40.65/465	NP_566071.1 GH3.11/JAR1/FIN219	39.97/450
GH3	XP_024543479.1 (annotated as GH3.12)	*Physcomytrium patens* XP_024386895.1 JAR1-like	37.69/294	NP_001319858.1 GH3.10/DFL2	35.64/591	NP_566071.1 GH3.11/JAR1/FIN219 #4: NP_001330076.1 GH3.12	34.89/258 35.29/249
UGT	XP_024542897.1	*A. thaliana* x *A. arenosa* KAG7593547.1	35.16/276	NP_173652.1 UGT85A7	33.74/273	NP_173656.1 UGT85A1 #8: NP_181910.1 UGT74F2	33.61/266 30.30/210
UGT	XP_024532226.1	*Picea sitchensis* ABR16170.1	35.70/299	NP_173652.1 UGT85A7	33.20/275	NP_173656.1 UGT85A1 #10: NP_187742.1 UGT76B1	32.12/267 32.42/221
UGT	XP_002992501.2	*Ginkgo biloba* ASK39407.1	37.86/300	n/a		NP_173656.1 UGT85A1 #17: NP_973682.1UGT74F1	34.77/270 29.35/203
UGT	XP_024526026.1	*Ziziphus jujube* XP_015865855.1	37.25/298	n/a		NP_173656.1 UGT85A1 #15:NP_187742.1 UGT76B1	37.16/290 29.71/219

^1^ n/a indicates that the top BLAST hit in Arabidopsis RefSeq database is the functionally characterized protein.

## Data Availability

Not applicable.
